# Supplementary material for: Complex‐centric proteome profiling by SEC‐SWATH‐MS
Source: Mol Syst Biol. 2019 Jan 14;15(1):e8438. doi: 10.15252/msb.20188438 (PMC6346213; doi:10.15252/msb.20188438)
Supplement: Supplementary file 8 — Dataset EV7 [file MSB-15-e8438-s008.zip › feature_plots_string/P61923.pdf]

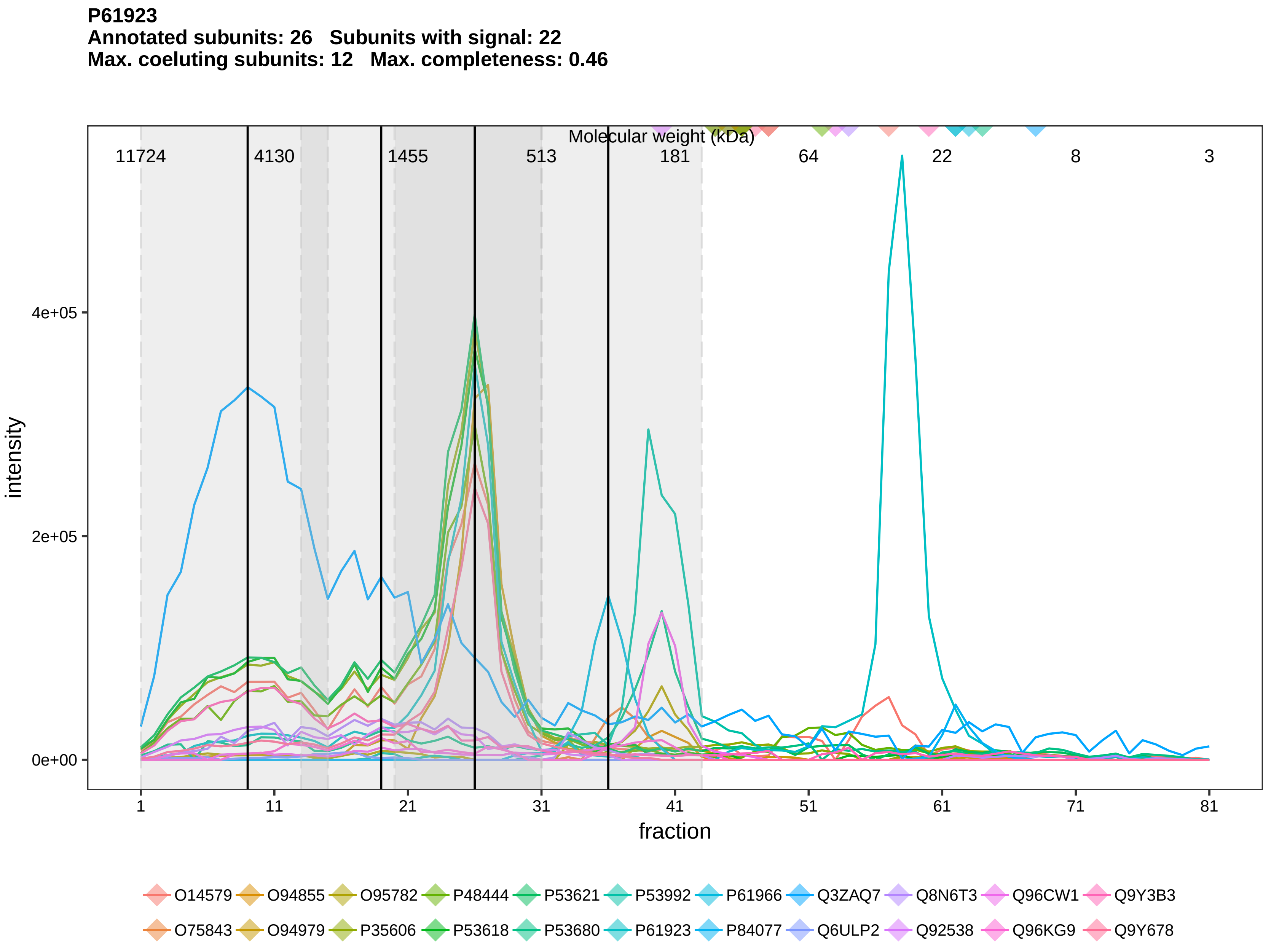

P61923
Annotated subunits: 26 Subunits with signal: 22
Max. coeluting subunits: 12 Max. completeness: 0.46
Molecular weight (kDa)
11724
4130
1455
513
181
64
22
8
3
intensity
4e+05
2e+05
0e+00
fraction
1
11
21
31
41
51
61
71
81
O14579
O94855
O95782
P48444
P53621
P53992
P61966
Q3ZAQ7
Q8N6T3
Q96CW1
Q9Y3B3
O75843
O94979
P35606
P53618
P53680
P61923
P84077
Q6ULP2
Q92538
Q96KG9
Q9Y678
